# Correlates of Protection for Cholera

**DOI:** 10.1093/infdis/jiab497

**Published:** 2021-10-20

**Authors:** Anita S Iyer, Jason B Harris

**Affiliations:** 1 Division of Infectious Diseases, Massachusetts General Hospital, Boston, Massachusetts, USA; 2 Department of Medicine, Harvard Medical School, Boston, Massachusetts, USA; 3 Department of Pediatrics, Harvard Medical School, Boston, Massachusetts, USA

**Keywords:** cholera, cholera vaccines, correlates of protection

## Abstract

A correlate of protection (CoP) is a measured adaptive immune response to vaccination or infection that is associated with protection against disease. However, the degree to which a CoP can serve as a surrogate end point for vaccine efficacy should depend on the robustness of this association. While cholera toxin is a dominant target of the human antibody response to *Vibrio cholerae* infection, antitoxin responses are not associated with long-term immunity, and are not effective CoPs for cholera. Instead, protection appears to be mediated by functional antibodies that target the O-polysaccharide coated *V. cholerae* outer membrane. Vibriocidal antibodies, which are complement-dependent bactericidal antibodies, remain the most accepted CoP for cholera and are used as surrogate end points in some vaccine studies. However, the association between vibriocidal antibody titers and immunity is not absolute, and they are unlikely to reflect a mechanistic correlate of protection against cholera.

Experts and regulatory bodies have proposed varied nomenclature to define correlates of protection (CoP; Table 1) [1–3]. In general, CoPs are measures of adaptive immunity (often antibody titers) that are associated with protection against either infection or disease and acquired by immunization and/or natural infection [4, 5].

**Table 1.** Terminology to Describe Correlates of Protection (CoPs) [2, 3]

Term	Definition
CoP	Immune response to vaccination or infection that is statistically related to protection against infection
Absolute CoP	Specific threshold level of a response at which there is a universal association with protection
Mechanistic CoP	A CoP that is causally responsible for protection
Nonmechanistic CoP	A CoP that is not causally responsible for protection

Most discussion of CoPs has focused on their use as surrogate end points for vaccine efficacy in clinical trials. However, there are no universally accepted standards that dictate the level of evidence required for CoPs to serve as trial end points. As a result, the acceptance of CoPs as proxies for vaccine efficacy by regulatory agencies, such as the US Food and Drug Administration and World Health Organization, depends on several factors.

For example, in the case of very rare infectious diseases, CoPs may be the only practical means to approximate vaccine efficacy. CoPs may also serve as surrogate end points in bridging studies for vaccines that have well-established efficacy. For example, CoPs can be used to compare minor changes in vaccine formulation, dosing regimens, and to make approximate comparisons of vaccine efficacy across in different populations. In contrast, an extremely high level of evidence linking a CoP with immunity, ideally including a clear mechanistic association, should be required for CoPs when used as surrogate end points for efficacy in first-of-kind vaccine trials. In addition, the use of CoPs as a surrogate clinical end point for vaccine efficacy to reduce the sample size required may not be beneficial when a large number of trial participants is needed to evaluate vaccine safety and rare adverse events [1].

In addition to their use as surrogate end points for vaccine trials, the identification of CoPs can serve other objectives. For some diseases, like hepatitis B or rabies, high-risk or potentially exposed individuals lacking evidence of protective immunity based on well-established cutoffs can be identified for additional vaccines or interventions [2]. Identifying CoPs for infectious diseases also can advance our understanding of the mechanisms of immunity and provide fundamental insights into host-pathogen interactions.

## COPS FOR CHOLERA

Oral cholera vaccines (OCVs) are increasingly being used and are a pillar of the World Health Organization’s strategy to reduce the burden of cholera by 90% by 2030 [6]. During the 15 years prior to the creation of a global cholera vaccine stockpile in 2012, only 1.5 million OCV doses were deployed, and in 2018 the number of doses used increased to over 17 million doses per year [7]. The benefits of vaccination include direct and indirect protection. A meta-analysis of 13 clinical trials and observational studies found that the average 2-dose efficacy of killed OCVs was 58% (95% confidence interval [CI], 42%–62%) [8]. Furthermore, in a large OCV trial in Bangladesh, in areas where vaccine coverage was >50%, there was an approximately 5-fold reduction in cholera incidence in placebo recipients compared to areas where coverage was <28% [9], demonstrating significant herd immunity in this setting.

Despite their benefits, current cholera vaccines are imperfect. Improving on their approximately 60% level of direct protection would be ideal. This is especially true in young children in whom direct protection from current OCVs is limited [10]. In addition, understanding the optimal delivery and dosing of OCVs are urgent practical questions. At a translational level, the identification of robust CoPs for cholera would help answer questions regarding the ideal formulation and dosing strategy for OCVs.

## PROTECTIVE IMMUNITY AGAINST *Cholera*

### Immunity Following Infection

Infection with *V. cholerae* O1 results in protection against subsequent disease. Human challenge studies, referred to also as controlled human infection models, in US volunteers demonstrate that a single episode of controlled classical *V. cholerae* O1 infection results in protection against reinfection for at least 3 years [11]. These models are corroborated by observations in cholera-endemic areas. For example, from 1991 to 2000, in an endemic area of Bangladesh, an episode of cholera conferred 65% protection (95% CI, 37%–81%) against subsequent hospitalization relative to age-matched controls. Infection with serotype Ogawa conferred homologous protection, while infection with serotype Inaba was associated with protection against both serotypes [12].

In individuals with no prior exposure, innate immunity provides a first line of defense against *V. cholerae*. Bacteria must survive passage through the acidic environment of the stomach, and then organisms must penetrate the mucus layer of the small intestine to reach the crypts [13]. These steps provide innate resistance to infection, and account for the high inoculum (approximately 10^8–11^) of bacteria required to cause infection [14]. However, once through this bottleneck, *V. cholerae* colonizes the crypt epithelium, multiplies, aggregates, and produces cholera toxin. In response, mucosal host defense proteins and oxidases are expressed at the epithelial surface, and cytokine-signaling pathways recruit immune cells to the lamina propria, including lymphocytes and neutrophils [15, 16]. These responses are likely central in shaping the adaptive immune response [17], and may be involved in clearing infection, but they are not sufficient to prevent disease. Almost all immunologically naive individuals who ingest enough bacteria will acquire disease. Similarly, as little as 5 µg of toxin delivered to the intestinal mucosa reproduces the symptoms of cholera [18]. These findings underscore the requirement for adaptive immunity in protection against cholera.

### Antibody-Mediated Immunity to Cholera

Antibodies have been a major focus of past research on adaptive immunity to cholera, and measures of circulating antibodies are the basis of most established CoPs [4]. Understanding the antigenic repertoire of infection is important in the identification of optimal CoPs. Interestingly, despite the thousands of proteins, sugars, and lipids made by *V. cholerae*, just a few antigens dominate the human B-cell response to cholera. In fact, when we evaluated a panel of antibodies produced by clonally expanded plasmablasts at the single cell/single antibody level, over 75% of these antibodies targeted 1 of 2 antigens: either cholera toxin or the O1-polysaccharide [19].

Although the focused repertoire of dominant antigens is an advantage in our understanding of immunity against cholera, the physical site of *V. cholerae* infection presents a formidable obstacle. Because *V. cholerae* is noninvasive, the secretion of antibodies into the intestinal lumen is a functional requirement for protection. While measures of circulating antibody responses are the basis of most established CoPs, mucosal antibody responses at the small intestinal surface are not easily measured and are not practical surrogate markers in clinical studies [20].

### Cholera Toxin Responses

Although antitoxin responses dominate the B-cell response to cholera, and antibodies are capable of neutralizing cholera toxin, these responses do not result in long-term protection against subsequent disease. Enteral immunization with cholera toxoid results in short-term decreases in diarrheal volume following challenge, but not significant protection [21]. Similarly, adding recombinant cholera toxin B-cell subunit (the receptor binding domain of the toxin) to killed whole-cell vaccines affords only a slight increase in protection, which lasts a few months after vaccination [22]. This may be because once *V. cholerae* colonizes the surface of the small intestine and begins to produce cholera toxin it is too late to mobilize neutralizing antibodies to the site of infection. Not surprisingly, serum levels of cholera toxin-specific immunoglobulin G (IgG) antibodies are a poor CoP for cholera [23–25]. Although high levels of serum cholera toxin IgA levels are a marker of protection in household contacts of individuals with cholera, these antibodies are very short lived after infection [24]. Similarly, the presence of circulating cholera toxin-specific memory B cells is not associated with protection after infection or vaccination. These finding are consistent with the observation of short-lived cholera toxin-derived protective immunity.

### O1 Polysaccharide and Functional CoPs

In contrast, antibodies directed against the *V. cholerae* O1-polysaccharide do appear to play a role in protection against subsequent cholera. This is underscored by an important observation: while many serogroups of *V. cholerae* exist in the environment, cholera is almost exclusively caused by a lineage of *V. cholerae* O1. However, when *V. cholerae* O139 emerged as a major cause of cholera from 1993 to 2002, through a single horizontal transfer of the *rfb* locus, previous infection with *V. cholerae* O1 did not confer protection against O139 [26], suggesting immunity is serogroup specific (although a caveat is that the capsule produced by the O139 strain could also obscure other cell surface targets). But while protective antibody responses likely target the O-polysaccharide, how they do this remains a key question for identifying optimal CoPs for cholera.

One problem is that not all measures of O1-antibodies appear to be equal CoPs for cholera. For example, increasing amounts of circulating IgA, IgM, and IgG antibodies that target the O1-polysaccharide are all highly correlated and associated with protection in household contacts of patients with cholera in Bangladesh [27]. However, the serum vibriocidal antibody titer, a measure of functional O1 antibodies, is a more robust biomarker of recent infection and a more robust CoP than the titer of circulating IgA, IgM, and IgG antibodies against the O1 antigen [27, 28].

This is likely because function of an antibody is not only determined by its antigen-binding domain, but also by structural interactions with the host innate immune system. These interactions are determined by the “tail” or Fc-region of the antibody. Fc-based interactions are determined by the anitbody isotype, subclass, and further determined by posttranslational modifications such as glycosylation [29]. Not surprisingly then, the serum vibriocidal antibody titer, which measures the concentration of antibodies that are capable of complement-dependent bactericidal activity and is mostly comprised of O1-antigen–binding IgM antibodies, is a more robust CoP than serum or fecal IgA, IgG, and IgM antibodies against *V. cholerae* [23, 24]. To restate this, while vibriocidal antibodies can be almost entirely depleted by removing antibodies that target the O1 polysaccharide [30, 31], the vibriocidal titer remains a more robust measure of protection than measures of total anti-O1 polysaccharide antibodies, likely because this measure also accounts for other determinants of antibody function.

## THE LIMITATIONS OF VIBRIOCIDAL ANTIBODY TITERS AND IDENTIFYING BETTER COPS FOR CHOLERA

As a result of its consistent association with protection, the vibriocidal antibody remains the most accepted CoP for cholera [23, 24, 32, 33]. Vibriocidal antibody responses have been utilized repeatedly as a primary end point in clinical bridging studies, which have had important regulatory implications [34–39]. But for good reason, major agencies like the Food and Drug Administration continues to require clinical trials measuring vaccine efficacy for new cholera vaccine formulations. And while vibriocidal antibody titers remain the best-established CoP for cholera, the lack of a mechanistic connection between the vibriocidal titer and protection is notable. There is no threshold vibriocidal titer at which 100% protection against cholera is observed [40], and complement-dependent bactericidal activity is unlikely to be a mechanism of protection against *V. cholerae* given the lack of evidence of the activation of the terminal components of the complement cascade at the small intestinal surface.

One possible approach to identify better CoPs for cholera is to consider the mechanisms by which antibodies could block colonization. For example, anti-O1 polysaccharide antibodies can agglutinate *V. cholerae* and dramatically inhibit motility even at subagglutinating concentrations, resulting in protection against cholera in animal models [41–43] *V. cholerae* also induces neutrophil efflux into the lumen and organisms can be cleared by NETosis in animal models [44]. It is also conceivable that antibodies may trap motile *V. cholerae* by anchoring them in the intestinal mucous [13].

Another, less-targeted approach to identifying better CoPs for cholera is to evaluate as many functional and nonfunctional antibody measures as possible to ask, when all possible markers of immunity are considered which ones are the most robust CoPs? This systems-serology approach [45] allows for an evaluation of antibody responses to a large number of antigens simultaneously and enables a holistic examination of the biophysical properties of antigen-specific antibodies such as glycan profiles, FcR-binding, antibody avidity, and resulting antigen-specific effector antibody functions [45]. An advantage of this approach is that it is less biased by mechanistic assumptions than a targeted evaluation.

A systems serology approach is also advantageous in assessing the relative contributions of multiple CoPs (or cocorrelates). Employing a principal component analysis or other machine learning methods can identify markers or combination of markers are best at distinguishing between protected and susceptible individuals. This in turn could lead to the identification of new CoPs that provide unexpected mechanistic insights into how antibodies might protect against cholera [45–47]. When using this approach to identify better CoPs against cholera, our initial findings underscore the value of this method. While many markers are in fact associated with protection, more work is needed to determine which CoPs stand out above the crowd in differentiating susceptible from protected individuals.

## EARLY COPS AND LATE COPS

The ideal CoP would perfectly distinguish protected from susceptible individuals at all time points after infection or vaccination. However, without a direct window to the small intestine, we are unlikely to find a perfect CoP for cholera. This is especially true when we are looking at serum antibody levels, and even if we could easily measure mucosal antibody levels in the small intestine there is good reason to believe that these would also not be an ideal longer-term CoP.

This is because there is evidence that immunity to cholera is maintained by the anamnestic immune responses of memory B cells. Memory B cells can rapidly differentiate into antibody-secreting cells upon exposure and are important in maintaining long-term immunity, but these cells are more difficult to measure than circulating or fecal antibody titers. In fact, the presence of circulating *V. cholerae* O1-polysaccharide–specific memory B cells is associated with protection against cholera even in individuals who have low antibody titers [48, 49]. Notably, in vaccinees who are challenged with live *V. cholerae*, vibriocidal antibody responses at day 10 after vaccination perform very well as a CoP at both day 10 and day 90 challenge, but vibriocidal antibody levels at day 90 are a poor CoP at day 90 compared to the presence of detectable O1-polysaccharide memory B cells at that late time point [49]. In addition, early (day 10) vibriocidal antibody responses were strongly predictive of a subsequent memory B-cell responses in this cohort [49]. This shows that with CoPs, the time at which the immune response is measured relative to the time at which protection is realized is a key consideration, although early CoPs are more practical proxy measures of vaccine efficacy in clinical trials than late measures.

## CONCLUSIONS

The vibriocidal antibody remains the current best accepted CoP for cholera. However, it not an absolute CoP, and it is unlikely there is a mechanistic association between the vibriocidal antibody response and protective immunity. Instead, it appears that an early vibriocidal antibody response against cholera is strongly predictive of the ability to generate a functional O1-antibody response that is maintained over a longer period of time, perhaps in the memory B-cell compartment. While a better mechanistic understanding of protection against cholera may lead to better CoPs, a systems-serology approach to identify the best CoPs may also lead to a better mechanistic understanding of immunity to cholera. Regardless of how we get there, the identification of better CoPs for cholera will help us to use existing cholera vaccines more effectively and advance the next generation of cholera vaccines.

## Notes

***Disclaimer.*** The funding source had no role in the writing or content of this manuscript.

***Financial support.*** This work was supported by the National Institutes of Health (grant numbers AI103055 and AI099243 to J. B. H.).

***Potential conflicts of interest.*** Both authors: No reported conflicts of interest. Both authors have submitted the ICMJE Form for Disclosure of Potential Conflicts of Interest. Conflicts that the editors consider relevant to the content of the manuscript have been disclosed.

Presented in part: Asian Conference on Diarrhoeal Disease and Nutrition, Dhaka, Bangladesh, 2020.
